# Optical Bio-Inspired Synaptic Devices

**DOI:** 10.3390/nano14191573

**Published:** 2024-09-29

**Authors:** Pengcheng Li, Kesheng Wang, Shanshan Jiang, Gang He, Hainan Zhang, Shuo Cheng, Qingxuan Li, Yixin Zhu, Can Fu, Huanhuan Wei, Bo He, Yujiao Li

**Affiliations:** 1 School of Integrated Circuits, Anhui University, Hefei 230601, China; 2 School of Materials Science and Engineering, Anhui University, Hefei 230601, China; 3 Yongjiang Laboratory (Y-LAB), Ningbo 315202, China

**Keywords:** optoelectronic, all-optical modulation, synaptic devices, neuromorphic computing

## Abstract

The traditional computer with von Neumann architecture has the characteristics of separate storage and computing units, which leads to sizeable time and energy consumption in the process of data transmission, which is also the famous “von Neumann storage wall” problem. Inspired by neural synapses, neuromorphic computing has emerged as a promising solution to address the von Neumann problem due to its excellent adaptive learning and parallel capabilities. Notably, in 2016, researchers integrated light into neuromorphic computing, which inspired the extensive exploration of optoelectronic and all-optical synaptic devices. These optical synaptic devices offer obvious advantages over traditional all-electric synaptic devices, including a wider bandwidth and lower latency. This review provides an overview of the research background on optoelectronic and all-optical devices, discusses their implementation principles in different scenarios, presents their application scenarios, and concludes with prospects for future developments.

## 1. Introduction

Traditional computers have had extraordinary achievements. AlphaGo, a “deep thinking” Go robot developed by Google, won the game against the world-famous player Lee Sedol in 2016, becoming the first robot to defeat the world champion of Go since IBM Deep Blue defeated Kasparov in 1997 [1]. Since then, artificial intelligence has gained more attention. However, the traditional von Neumann computer architecture has hindered its development speed. The fundamental principles of von Neumann architecture have remained unchanged until John von Neumann introduced the concept of stored programs in 1952 at the Institute for Advanced Study Machine [2]. Presently, CPU processing speeds significantly surpass memory access speeds, resulting in CPUs idling while waiting for data retrieval. Furthermore, this segregation between storage and computation structures also contributes to substantial energy consumption [3]. Nevertheless, with the emergence of the Internet of Things (IoT), traditional computers face increasingly daunting challenges posed by storage and power consumption bottlenecks inherent in von Neumann architecture [4]. 

**Figure 1:**
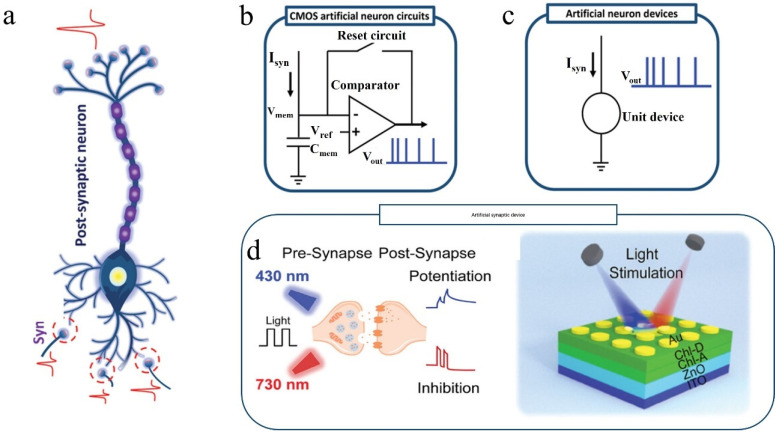
(**a**) Diagram of neurons and synapses. Spike signals are produced by postsynaptic neurons upon the integration of an adequate amount of signals from synapses. Schematic diagram of (**b**) CMOS artificial neuron circuits and (**c**) artificial neuron devices [12]. (**d**) An example of an artificial all-optical synapse [42].

In contrast, the human brain is arguably the most sophisticated system on Earth [5], excelling in processing diverse analog signals and integrating them into coherent images, storing certain memories for decades, and exhibiting remarkable reasoning capabilities [6]. It is worth mentioning that these functions only necessitate approximately 20 W of energy [7,8], which is roughly one-tenth of what a typical desktop computer consumes. With around 10^11^ neurons and 10^15^ synapses [9], the brain forms an incredibly intricate yet efficient network [10]. Neurons communicate through synapses, where neurotransmitters are released when signal strength surpasses a threshold, carrying vital information [11]. This process is shown in Figure 1a. The transmission strength between synapses relies on synaptic weights or connection strengths [12], with synapses themselves displaying plasticity to enable changes in synaptic weights to occur [13,14]. This unique phenomenon is absent in the conventional memory system. Drawing inspiration from this, numerous researchers have endeavored to develop artificial neurons that mimic information transmission processes within the brain (Figure 1b). Using traditional CMOS technology will require multiple comparators and capacitors. Nonetheless, high-value capacitors are costly in standard CMOS processes [15], and comparators occupy substantial area space. Therefore, CMOS devices do not offer advantages in terms of energy efficiency or scalability [16] because CMOS devices were not originally developed to simulate neurons. Figure 1c depicts an ideal model where just one unit can emulate a neuron; such an advancement would be groundbreaking, both in terms of power consumption and spatial coverage. The pursuit of achieving the large-scale deployment of neural morphologies necessitates the collaborative efforts of synapse devices, axon devices, and dendrite devices. Currently, there exist silicon-based CMOS analog synapses, exemplified by IBM’s TrueNorth chip [17] and Intel’s Loihi chip [18]. In recent years, there has been a proliferation of neuromorphic computing devices, such as various three-terminal transistors and two-terminal memristors [19,20,21,22,23,24,25,26,27], capable of achieving in-memory computation. This capability is particularly advantageous for overcoming the von Neumann memory wall. By applying different stimuli to these devices, they exhibit diverse responses that resemble real neural synapses, thus earning the designation of artificial neural synapses. The strength of connections between synapses can be quantified by measuring the conductivity between devices [28,29,30]. These artificial synapses offer promising hardware foundations for the advancement of neuromorphic computing chips. However, most existing artificial synapses primarily rely on electrical stimulation, which inevitably imposes limitations on their operational speed and bandwidth due to inherent device constraints [31,32,33,34,35]. Introducing light as a stimulus presents an effective solution to this predicament since light boasts high bandwidth, low crosstalk, low power consumption, and no RC delay [33,36,37].

Figure 1d is an example that demonstrates the device’s response to light by overlaying different functional layers. A superior artificial synapse should not only accurately simulate synaptic behavior, but also possess characteristics such as low power consumption, low operating voltage, excellent durability, linear conductivity rise and fall, and good symmetry [38,39,40,41]. These requirements also apply to optoelectronic synapses and all-optical control synapses, presenting challenges in material selection and coordination among different functional layers.

In recent years, more and more researchers have tried to introduce light into artificial synapses. These artificial synapses involving light stimulation can be classified into two categories: optoelectronic artificial synapses and all-optical artificial synapses. For the former, excitatory synapses are elicited by light, while inhibitory synapses still require electrical pulse stimulation [43,44,45]. For the latter, both excitatory and inhibitory synapses can be elicited by light. Recently, numerous all-optical artificial synapses have emerged, for instance, those employing IGZO/SnO/SnS heterostructure [46], Ag-TiO_2_ nanoclusters/sodium alginate film [47], photochromic perovskites [48], and phase change material [49]. The all-optical synapse can fully exploit the advantages of light because there is no involvement of electrical stimulation. Compared with the optoelectronic synapse that requires a combination of light and electrical stimulation, the operation of the all-optical synapse is simpler, as the experimenter does not need to consider too many issues of coordinating light and electricity. In the all-optical synapse, a suitable small voltage bias is usually applied to both ends of the device (many articles refer to this as the read voltage), and the subsequent operation will not involve any electrical components. Currently, there have been studies that suggest this separation operation model can reduce device power consumption, minimize the damage caused by Joule heat to the device microstructure, and help to solve the issue of device stability [43]. In view of the many related papers published in recent years, it is necessary to summarize these optical bio-inspired synaptic devices, explore the physical explanations behind the synaptic functions that these devices can achieve, and explain their practical applications. 

This review mainly summarizes the recent research results of optoelectronic synapses and all-optical synaptic devices, along with their principles. Section 3 is about optoelectronic artificial synapses and Section 4 is about all-optical artificial synapses, followed by materials (Section 5), applications (Section 6), and future challenges (Section 7), and the second chapter will introduce some basic knowledge about neural synapses and how they are manifested in devices. As mentioned earlier, all-optical artificial synapses have more advantages compared to optoelectronic artificial synapses, and considering the lack of comprehensive reviews on all-optical artificial synapses at present, this article will focus more on introducing all-optical artificial synapses. This review will help researchers who are about to engage in artificial synapses to understand this field more efficiently. At the same time, it helps researchers who have carried out research in this field by summarizing findings and inspiring them.

## 2. Basic Synaptic Functions and Device Simulation Methods

Before delving into further discussion, it is imperative to comprehend the fundamental functionality of synapses and how they are verified in artificial synapses devices. The experimental curves derived from artificial synaptic light or electrical stimulation should exhibit a resemblance to the response curves observed in biological synapses found in nature. This concept aligns with the principles of “bionics”. The notion that structure governs function [50,51] applies universally to both synapses and circuits.

### 2.1. Postsynaptic Current Responses

**Figure 2:**
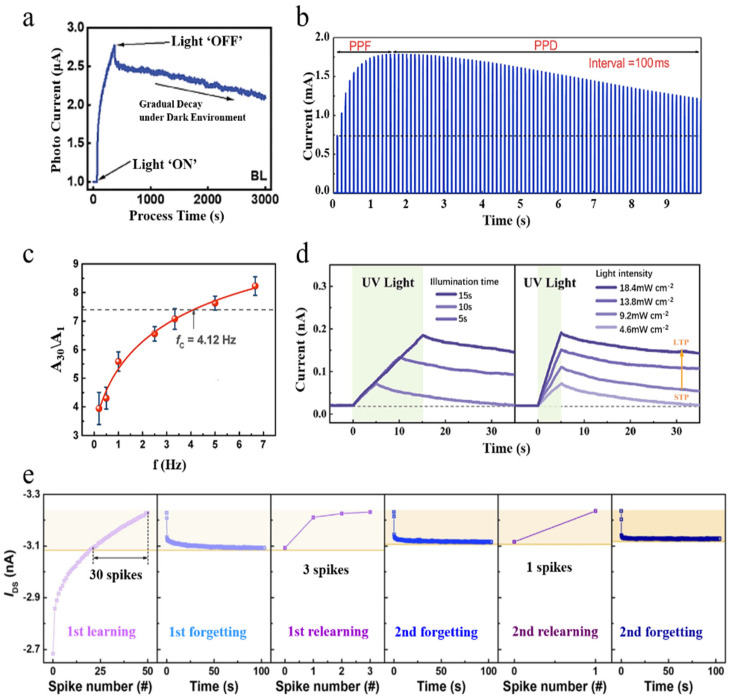
(**a**) Photoresponse of bilayer device [55]. (**b**) In the C_3_N/PVPy-based memristor, continuous stimulation can result in both post-potentiation facilitation (PPF) and post-potentiation depression (PPD) [56]. (**c**) Frequency response of artificial synapses; here, A30/A1 is defined as the gain [57]. (**d**) Test results of PSC responses to stimuli of different light intensities and light durations in a device fabricated based on ZnO/MoO_x_ [58]. (**e**) The learning–forgetting–relearning experiment [59].

The synaptic weight is extremely important, as mentioned earlier in this article, as it plays a crucial role in the transmission of information between neurons. In biological systems, the ability of synaptic weight to be modulated by neural activity is called synaptic plasticity, and it is the basis of various aspects of complex learning, cognition, and memory. Many properties of artificial synapses are tested based on synaptic weight. Currently, almost all of the literature defines the conductivity or resistance of the device as the synaptic weight, and according to Ohm’s law, the magnitude of the resistance/conductance can be reflected by applying voltage. During testing, a certain reading voltage is applied to the artificial synapse, followed by different stimuli (in the form of light or electricity), and then the response current (referred to as postsynaptic current) is measured. The carrier can be considered a neurotransmitter [52]. By analyzing peak currents, rates of increase and decay, final stable values, power consumption, etc., and further analyzing changes in conductivity, we can obtain information about the properties of the artificial synapse being tested and evaluate it accordingly, just as the PSC of real synapses is analyzed to obtain their properties. Synaptic plasticity is demonstrated when the current does not return to the initial value for a short time after the device is stimulated by electricity or light. Figure 2a shows common PSC curves that are frequently seen in papers related to artificial synapses. Postsynaptic current (PSC) can also be divided into inhibitory PSC (IPSC) and excitatory PSC (EPSC) [53], and in three-terminal devices, PSC can also be controlled by gate electrodes. Li et al. have utilized this feature to control the magnitude of device response current for better adaptability to environmental conditions [54].

### 2.2. STP and LTP Behaviors

According to the temporal relationship between synaptic connection strength and time, synaptic plasticity can be categorized into two distinct forms: short-term plasticity (STP) and long-term plasticity (LTP) [60]. The interconversion among STP, LTP, and their transitional states can be discerned by applying a reading voltage [61]. If the artificial synapse is in the LTP state, then this means that the artificial synaptic connection is strong; in other words, the conductance of the synapse is large. On the contrary, if the artificial synapse is in the STP state, then it means that the artificial synaptic connection strength is weak; in other words, the synaptic conductance is small. As shown in Figure 2d, the current through the artificial synapse in LTP was significantly larger at the same read voltage. STP exerts significant impacts on the control of motor functions, recognition of speech, and retention of information in working memory. On the other hand, LTP plays a crucial role in encoding spatial data. The coexistence of STP and LTP is believed to facilitate highly adaptable behavior and advanced cognitive abilities [60,62].

A significant test method for assessing STP levels is paired-pulse facilitation (PPF) and paired-pulse depression (PPD). PPF (PPD) requires the application of two consecutive stimuli, with the response generated by the second stimulus being stronger (weaker) than that generated by the first stimulus. Certain devices can test both PPF and PPD phenomena [56], as shown in Figure 2b. In biological synapses, the depolarization of the presynaptic membrane induces Ca^2+^ influx. If the time interval is sufficiently small, then the Ca^2+^ concentration is triggered by a preceding pulse can be enhanced by subsequent pulses, thereby increasing the probability of vesicle release, which defines PPF. On the other hand, PPD can occur due to either the inactivation of voltage-dependent Ca^2+^ channels or the temporary depletion of neurotransmitter vesicles in presynaptic neurons [56,63].

Long-term potentiation (LTP) is a persistent alteration in synaptic strength resulting from specific patterns of synaptic activity, serving as a cellular model for information storage in the central nervous system, and has garnered considerable attention [64]. From an artificial synapse perspective, LTP signifies that the device conductivity surpasses its initial level significantly.

### 2.3. Human Memory Behaviors

In the human brain, information is stored through the process of learning, and memory levels can be enhanced through repeated training [24,65]. This phenomenon is associated with the conversion from short-term potentiation (STP) to long-term potentiation (LTP). Taking paired-pulse facilitation (PPF) as an illustrative example, the response to the second stimulus exhibits increased strength; however, subsequently, there is a gradual decline in excitatory postsynaptic current (EPSC). A plausible speculation suggests that by applying more stimuli, the peak amplitude of EPSC would be higher while its rate of decay would be slower. Manipulating the frequency and quantity of stimuli may induce a transition from STP to LTP, which means that the synaptic weight may increase with the frequency and intensity of the stimulus. In terms of memory, this means that increasing stimuli may make our memories stronger. While these points are obviously true from a common-sense perspective, the researchers did the following experiment for the sake of rigor.

Many researchers conduct relevant experiments to determine the efficacy of their created devices in simulating memory processes. Zheng et al. developed a full-optical control device based on ZnO/MoO_x_ heterojunction structure, successfully inducing a transition from a low-conductivity state to a high-conductivity state by manipulating the time and intensity of the applied light stimulation, thereby achieving the transition from short-term potentiation (STP) to long-term potentiation (LTP) [58]. If we consider postsynaptic currents (PSCs) as indicators of memory levels, then Figure 2d can also be interpreted as repeated stimuli enhancing the robustness of memories, which aligns with common knowledge. This can be verified by altering the rate and number of applied stimuli [66,67,68]. 

On the other hand, since the synaptic weight rises with the frequency of the stimulus, it is expected that the gain of EPSC will rise with the synaptic weight. In other words, the synapses have the characteristics of high-pass filters. Some papers also use the method of measuring gain–frequency plots to prove that their devices can simulate memory functions [69,70]. In addition, due to the high-pass filtering property of synapses, some papers try to explain the application of artificial synapses in signal processing [57]. As depicted in Figure 2c, the gain does increase with the frequency (the gain of the device is defined as EPSC after applying 30 pulses at each frequency divided by EPSC after applying the first pulse).

Partial memory information in humans tends to be forgotten over time, which corresponds to the device transitioning from a highly conductive state after stimulation back to a low-conductive state. However, relearning forgotten knowledge requires less effort and time. This is the learning–forgetting–relearning experiment, which has been reported in several papers on artificial synapses [71]. Figure 2e clearly illustrates this process, showing that while 30 pulses are required for initial learning, only 1 pulse is needed for subsequent learning [59].

## 3. Optoelectronic Artificial Synapse

The history of optoelectronic artificial synapses can be traced back to the early 21st century. Pina et al.’s work in 2000 demonstrated that specific solutions could integrate ultraviolet light through chemical reactions [72]. Importantly, this early research established the influence of light on synapses. Since 2016 [73], optoelectronic artificial synapses have gained significant popularity as a research focus. Currently, reported optoelectronic artificial synapses employ diverse materials and structures based on different principles, each offering unique advantages. This section will elucidate the fundamental principles pertaining to photoelectric synapses. It is crucial to emphasize that, regardless of the presence or absence of light, the primary objective of artificial synapses remains the regulation of conductance, as it directly signifies the synaptic weight.

### 3.1. The Formation and Breakage of Conductive Filaments

**Figure 3:**
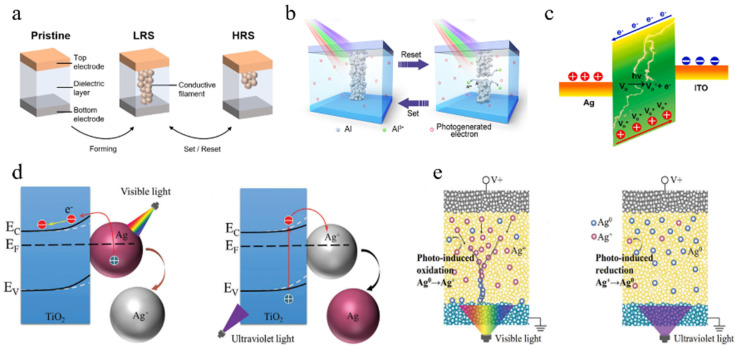
(**a**) For an artificial synapse with a sandwich structure, the formation (breaking) of conductive filaments implies a SET (RESET) operation, and the device conductance rises (falls) [74]. (**b**) The occurrence of conductive filaments takes place in the state of low resistance (LRS), whereas the disruption of conductive filaments happens in the state of high resistance (HRS) [23]. (**c**) Energy band diagram of an optoelectronic memristor based on ITO/ZnO/Ag structure under illumination conditions [75]. (**d**,**e**) Schematic illustrations depicting the processes of synaptic modification induced by light and synaptic modification driven by electrical stimulation, respectively [76].

The formation and fracture of conductive filaments represent a prevalent mechanism, wherein the presence of an uninterrupted conductive filament between positive and negative electrodes results in high conductivity, while its absence leads to low conductivity. Figure 3a depicts a conventional non-optical artificial synapse based on conductive filaments, where the creation and disconnection of the filament are achieved by applying voltage with different polarities to the electrodes. Additionally, there have been reports suggesting that thermal-assisted electrochemical processes can induce fracture during the process of conductive filament rupture [74].

For optoelectronic artificial synapses, a viable approach involves utilizing light to regulate the process of conductive wire fracture or conduction, or to create conditions for the formation of conductive wires and indirectly control their development. Liu et al. [23] fabricated an artificial synapse with a glass/ITO/TiS_3_/Al structure. During fabrication, Al conductive wires were present, resulting in the high electrical conductivity of the initial device. The oxidation and reduction of these Al conductive wires can be achieved by applying different polarities of voltage. Oxidation leads to wire fracture while reduction allows for wire reformation. As depicted in Figure 3b, when illuminated, TiS_3_ generates numerous photogenerated charge carriers that migrate near the Al conductive wires and inhibit their oxidation. Consequently, after illumination, the device maintains a higher conductivity state—a typical example where light suppresses conductive wire fracture.

Wang et al. [75] developed an artificial synapse comprising an ITO/ZnO/Ag structure. The energy band diagram is illustrated in Figure 3c. When exposed to light, the functional layer excites multiple electron–hole pairs, resulting in electrons attracting Ag^+^ ions from electrodes into this layer. This process facilitates conducting wire formation and enables a transition from low conductivity to high conductivity—representative of how light controls conducting wire formation.

Shan et al. [76] created a structure consisting of Au/TiO_2_/FTO but incorporated Ag nanoparticles into TiO_2_. Figure 3d demonstrates how Ag nanoparticles are oxidized and reduced through exposure to light. Utilizing light-induced oxidation produces the necessary Ag^+^ ions required for forming conducting wires. This method eliminates the need for electric pulses as it employs ultraviolet light instead. It effectively reduces operating voltage and power consumption. This is an example of the indirect control of conductive filaments formed by light.

### 3.2. Barrier Type and Depletion Layer Type

When two materials with mismatched energy band structures come into contact, electron flow occurs primarily from one material to another due to disparities in work function and electron affinity, resulting in the bending of bands. The interface can potentially create a higher barrier and depletion layer, leading to reduced device conductivity. Different band alignments can govern carrier transport directionality. Illumination has the potential to induce ionization of vacancies, thereby modifying the energy band structure and subsequently influencing conductivity.

**Figure 4:**
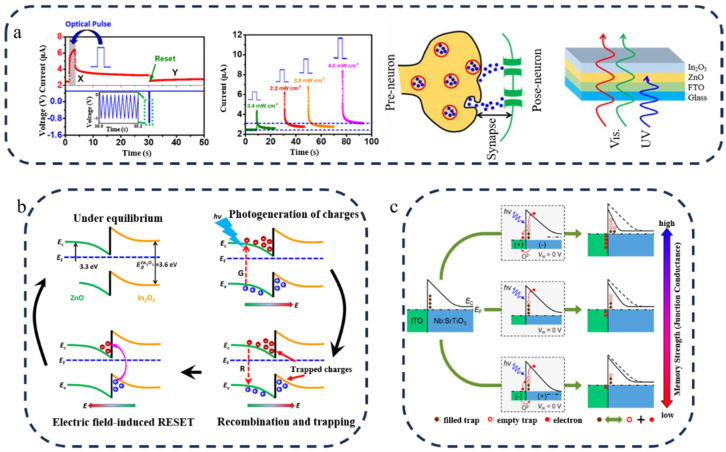
(**a**) Left: The PSC response curve of the device was obtained by first applying UV light, then turning off UV light, and then applying a −1 V pulse after 30 s, and current–time curves of the device after light stimulation of different intensities. Right: Device schematic diagram [77]. (**b**) Energy band diagram of photoelectric devices based on ZnO/In_2_O_3_ heterojunction [77]. (**c**) Energy band diagram of the artificial optoelectronic synapse based on the heterojunction between ITO and Nb:SrTiO_3_ [24].

Mohit Kumar et al. [77]. successfully achieved the properties of synapses by utilizing an In_2_O_3_/ZnO heterojunction. As depicted in Figure 4a, the application of ultraviolet light stimulation induced a change in the conductivity of the device, effectively mimicking synaptic plasticity. The underlying principle is elucidated in Figure 4b: when the light stimulus is removed, electrons and holes become trapped in ZnO and In_2_O_3_, respectively, due to the barrier presented by the heterojunction, thereby reducing the interface energy barrier. Gao et al. [24] fabricated a photonic synapse with a functional layer structure composed of ITO/Nb:SrTiO_3_, as illustrated in Figure 4c, which demonstrates its energy band structure and working mechanism. Owing to distinct energy band structures between ITO and Nb:SrTiO_3_, a barrier is formed that places the device into a high-resistance state. However, numerous defects at the interface capture many electrons; thus, illumination releases these electrons, resulting in an abundance of positively charged traps at the interface that elevate its potential while bending down its energy band structure and lowering its barrier, consequently enhancing electron tunneling probability. This ultimately leads to an increase in device conductivity.

### 3.3. Three-Terminal Optoelectronic Transistor

In addition to the utilization of two-terminal devices, optoelectronic synapses can also be implemented using three-terminal devices. The key characteristic of three-terminal devices lies in their controllability through the gate, achieved by applying varying voltages or inducing different potentials on the gate, thereby influencing the conductivity of the channel.

**Figure 5:**
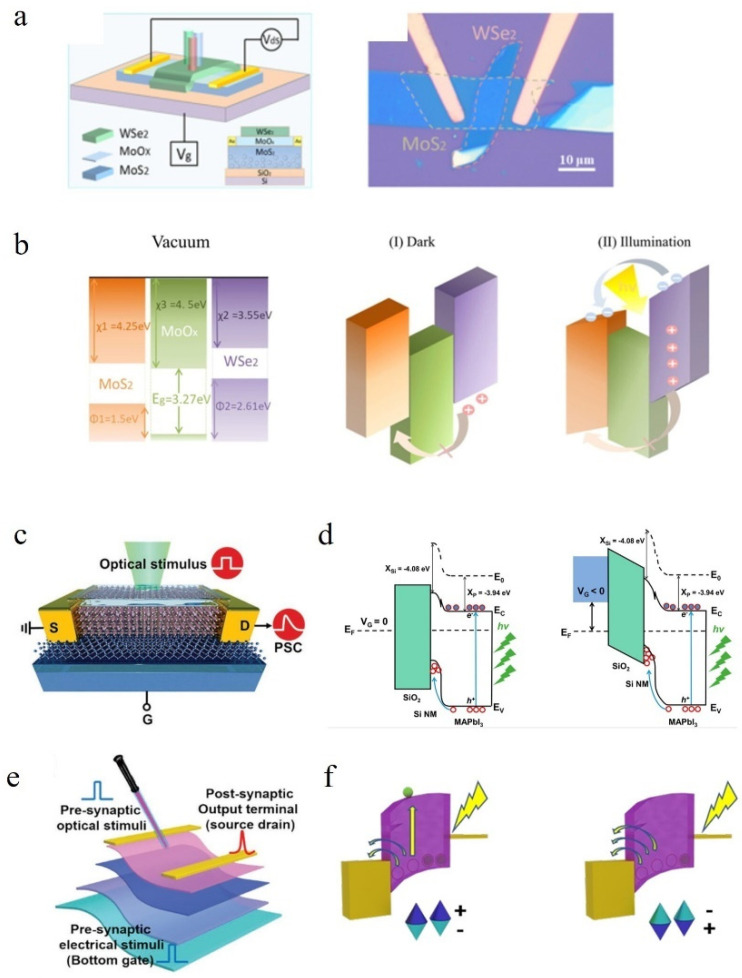
(**a**) Schematic diagram and optical microscopy image of floating gate transistor. (**b**) Energy band diagram under illumination and dark conditions. (I) Dark state and (II) illumination state [78]. (**c**) The synaptic transistor utilizing the hybrid composition of Si nanomembrane and MAPbI_3_ [81]. (**d**) In the scenario where the gate voltage is either equal to or less than 0, the energy band diagram of the device under light illumination [81]. (**e**) Schematic illustration depicting the light modulation of a flexible optoelectronic device [84]. (**f**) Schematic representation illustrating the energy band of the photoelectric dual-modulation transistor when subjected to optical illumination conditions [84].

For a three-terminal transistor, one of the most important parameters is the threshold voltage. By changing the threshold voltage, the conductivity resistance of the transistor can be altered, and a commonly used method is through a floating gate structure. To ensure that the floating gate layer carries the correct charge, careful design of its structure is required based on different materials’ energy band structures. Wang et al. have fabricated a floating gate transistor with device structure and band structure as shown in Figure 5a and Figure 5b, respectively. In this case, WSe_2_ serves as the floating gate layer, MoO_x_ acts as the tunneling layer, and MoS_2_ functions as the channel. Illumination generates numerous electron–hole pairs in WSe_2_; however, due to barrier blocking for holes, they become trapped in the floating gate layer, resulting in positive charge accumulation. The physical isolation between electrons and holes prevents their immediate recombination, thus simulating the long-term plasticity of synapses [78].

Heterojunctions are widely employed in optoelectronic synapses due to their ability to effectively separate electron–hole pairs generated by light, thereby influencing the energy bands. According to the arrangement of energy bands, heterojunctions can be categorized into three different types: gap straddling (type I), gap staggering (type II), and gap breaking (type III) [79]. Recent research has demonstrated that type II heterojunctions exhibit optimal performance for creating optoelectronic synapses, as the conductivity of the channel in this structure is highly sensitive to carrier separation and recombination [80]. Building upon this principle, Yin et al. [81] proposed a structure shown in Figure 5c, where a p-type Si NW and n-type MAPbl_3_ heterostructure with type II band alignment was constructed, successfully achieving synaptic functions such as EPSC, PPF, STP, and LTP, and the device exhibits high photosensitivity, resulting in reduced power consumption. In the presence of light, a significant number of electron–hole pairs are generated. The built-in electric field facilitates the migration of holes to the Si NW while electrons remain in the channel, resulting in an augmented electron concentration in the n-type channel and subsequently an increase in source-drain current, which serves as the foundation for EPSC. The heterojunction effect effectively hinders the immediate recombination of photogenerated electron–hole pairs, establishing the basis for LTP. Moreover, applying a negative bias to the gate electrode leads to further hole accumulation, thereby amplifying EPSC. The entire process is depicted in Figure 5d.

In recent years, there has been significant advancement in the development of ferroelectric materials, which exhibit spontaneous polarization at specific temperatures without requiring external power sources. Ferroelectric transistors exhibit impressive switching speeds, rendering them exceptionally suitable for applications with minimal power consumption [82,83]. Researchers utilize the inherent polarization of ferroelectric materials to effectively control charge carrier accumulation and depletion within the channel [82]. Figure 5d showcases a flexible ferroelectric transistor fabricated by Li et al., utilizing P(VDF-TrFE) as the ferroelectric material. The plasticity of synapses is achieved through the precise modulation of the remnant polarization exhibited by P(VDF-TrFE) [84]. As shown in the Figure 5f, when illuminated, a significant number of electron–hole pairs are generated in the conductive channel, while surface traps also induce a substantial influx of charge pairs into the channel. This phenomenon serves as the foundation for EPSC. The carrier concentration in the channel can be controlled by the polarization direction of ferroelectric materials, thereby resulting in a hysteresis curve for I_D_-V_G_ characteristics.

## 4. All-Optical Artificial Synapse

Research indicates that visual sources contribute to 80% of human information acquisition [85]. In a significant proportion of optoelectronic synapses, the enhancement of device conductivity is achieved through light stimulation; however, it often necessitates the application of reverse bias voltage to restore the initial state of device conductivity. In biological organisms, inhibitory neurotransmitters also exist [86], thus fostering the prospect of developing all-optical control synapses. In this paper, light capable of augmenting device conductivity (reinforcing synaptic connections) is denoted as SET light, while light with an opposing effect is referred to as RESET light.

The advantage of optoelectronic synapses lies in their ability to combine the benefits of light and electricity, resulting in simpler operation compared to all-optical devices [87]. However, it is important to note that operational simplicity can be compromised for performance enhancements. All-optical synaptic devices possess numerous exceptional properties such as nearly wireless bandwidth, support for parallel processing, high efficiency, low crosstalk, and real-time capability [88,89,90], making them highly appealing [91]. The currently reported all-optical synapses employ various principles including modulation based on interface potential barriers, oxidation–reduction reaction-based modulation, gas adsorption, desorption-based modulation, etc. The following section will introduce the fundamental principles of various all-optical synaptic devices that have emerged in recent years.

### 4.1. Barrier Type

When two semiconductors or a semiconductor and a metal come into contact, the difference in their band structures gives rise to the formation of a junction barrier. The structural characteristics of this junction barrier significantly impact electron tunneling probability, which is macroscopically observed as device conductivity. Recent studies have highlighted the crucial role of interface barrier modulation in all-optical synaptic devices. Barriers can be formed by PN junctions, heterojunctions, and metal–semiconductor contacts.

#### 4.1.1. PN Heterojunctions

**Figure 6:**
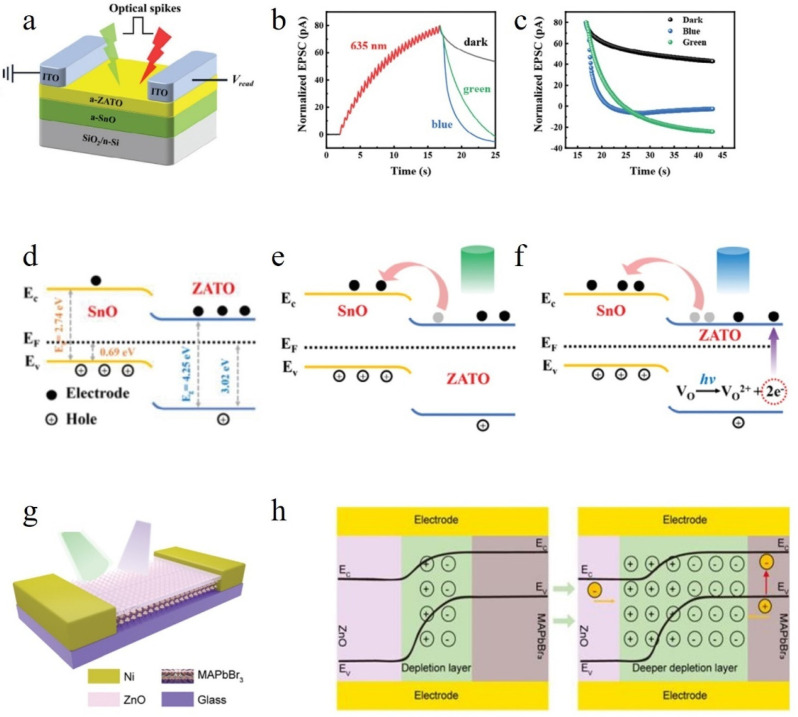
(**a**) The design of a two-terminal planar configuration for an all-optical controlled synapse utilizing amorphous ZnAlSnO/SnO materials [43]. (**b**) The variation of the current in different lighting environments [43]. (**c**) Decay curves of different optical environments [43]. (**d**) The diagram illustrating the energy band structure upon the contact between n-ZATO and p-SnO [43]. (**e**,**f**) The behavior of electrons when the device is irradiated with green light and blue light [43]. (**g**) The schematic of the synaptic device [92]. (**h**) The schematic illustration of perovskite–ZnO heterojunction before and after green light illumination [92].

As illustrated in Figure 6, Yang et al. fabricated a PN junction-based all-optical synaptic device employing n-ZATO and p-SnO [43]. Initially, the device was exposed to 30 red light pulses at 635 nm wavelength. Due to the high concentration of oxygen vacancies in amorphous oxide semiconductors, they exhibit sensitivity towards visible light, with red light ionizing these vacancies and resulting in an enhancement of device conductivity. Subsequently, the device was subjected to different illumination conditions to investigate the correlation between PSC and time, aiming to explore its potential for achieving optical suppression. The corresponding outcomes are depicted in Figure 6b,c. It is observed that when the device is exposed to blue or green light environments, its conductivity decay rate is accelerated compared to being placed in a dark environment. This observation suggests that short-wavelength light facilitates a reduction in conductivity. The band diagrams presented in Figure 6d–f elucidate this phenomenon. ZATO and SnO exhibit distinct responses towards incident light: Under green illumination, conduction band electrons from ZATO surmount barriers and enter SnO’s conduction band, leading to reduced conductivity, whereas blue light possesses higher photon energy than green light, which expedites this process. Throughout this mechanism, ZATO and SnO play pivotal roles by compensating for their disparate responses towards photons with varying energies as well as facilitating electron migration.

The depletion layer width is a critical parameter for a PN junction, as it determines the device’s characteristics. To modify the properties of the PN junction, different light sources are required for all-optical control, eliminating the need for electrical signals. Ge et al. successfully developed an all-optical controlled synapse based on a PN junction using MAPbBr_3_ perovskite and ZnO [92], as depicted in Figure 6g. They conducted 20 consecutive experiments and observed no degradation in performance while testing EPSC, IPSC, and PPF properties. The band structure is illustrated in Figure 6h. When exposed to ultraviolet light, ZnO exhibits strong absorption and generates numerous photogenerated holes that discharge chemisorbed oxygen ions while retaining photogenerated electrons, resulting in a positive photoresponse. Conversely, when green light irradiates ZnO, photogenerated electrons and holes diffuse deeper into the depletion layer region, causing reduced effective carrier transport and optical suppression [92].

#### 4.1.2. Multi-Layer Heterojunctions

**Figure 7:**
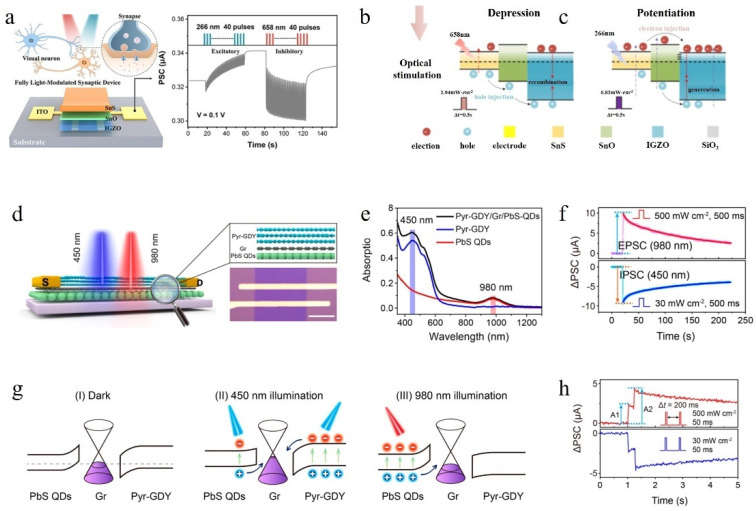
(**a**) Schematic illustration of the IGZO/SnO/SnS neuromorphic synapse structure [46]. (**b**,**c**) Energy band diagrams and carrier transfer activities of the IGZO/SnO/SnS synaptic device under different wavelengths of illumination [46]. (**d**) Characterizations and optoelectronic properties of an optical synapse utilizing a heterostructure composed of Pyr-GDY/Gr/PbS-QD [28]. (**e**) Optical absorption spectra for Pyr-GDY (blue), PbS QDs (red), and Pyr-GDY/Gr/PbS-QD heterostructure (black) [28]. (**f**) The device exhibits EPSC and IPSC when stimulated by an optical pulse with wavelengths of 980 nm and 450 nm, correspondingly [28]. (**g**) Energy band arrangement of the Pyr-GDY/Gr/PbS heterostructure in (**I**) absence of light, (**II**) under 450 nm light exposure, and (**III**) under 980 nm light exposure [28]. (**h**) PPF and PPD curves of all-optical synapse based on Pyr-GDY/Gr/PbS heterostructures [28].

The PN heterojunction functional layer mentioned above comprises two materials. To enhance its performance, the number of material layers in the functional layer can be increased. Zhang et al. successfully fabricated a fully optical synapse using a three-layer heterojunction consisting of IGZO, SnO, and SnS [46]. Figure 7a illustrates the device structure diagram, while Figure 7b presents the PSC results for this device under irradiation with 266 nm ultraviolet light and 658 nm light, respectively. Notably, in this device, the SET light corresponds to short-wavelength ultraviolet light, whereas the RESET light represents long-wavelength red light.

The band diagram of the device is shown in Figure 7c. When illuminated by 658 nm light, SnS generates electron–hole pairs, and under the influence of the built-in electric field, holes drift to the boundary and are injected into IGZO where they recombine with electrons, resulting in a decrease in electron concentration and a decrease in conductivity. When illuminated by shorter-wavelength light, both SnS and SnO generate electron–hole pairs that simultaneously enter IGZO. Holes arrive first, causing a decrease in PSC after irradiation with 266 nm light; however, throughout the entire illumination process, the excitation of electrons in IGZO exceeds recombination, leading to an overall increase in conductivity. The reason for using a three-layer heterojunction is that the middle layer of SnO effectively reduces the barrier that must be overcome for hole injection from SnS into IGZO, thereby improving injection efficiency. Additionally, the surface quality of the SnS/SnO heterojunction facilitates hole transport.

Hou et al. have developed a high-performance all-optical synaptic device based on a three-layer heterostructure of graphdiyne/graphene/PbS quantum dots [28]. The schematic diagram of the device is depicted in Figure 7d, showcasing its intricate structure. The design of the heterojunction relies on three key factors: appropriate band bending direction, material absorption for different wavelengths of light, and p-type doping in the central graphene channel. Figure 7e illustrates the absorption characteristics of various material combinations for distinct wavelength ranges. It can be observed that Pyr-GDY exhibits significantly higher absorption rates than PbS QDs in the short-wavelength range, while PbS QDs effectively absorb light at 980 nm wavelength, and Pyr-GDY shows minimal absorption. When subjected to irradiation with 980 nm and 450 nm light, respectively (as shown in Figure 7f,h), it becomes evident that SET operation utilizes 980 nm light while RESET operation employs 450 nm light. The band diagram presented in Figure 7g elucidates this phenomenon: upon exposure to 450nm light, both PbS QDs and Pyr-GDY absorb photons; however, due to stronger absorption by Pyr-GDY and direct illumination effect, an increased number of electron–hole pairs are generated. Consequently, under the influence of a built-in electric field, numerous electrons enter into the p-type graphene channel while only a few holes do so, resulting in decreased carrier concentration and reduced conductivity. Conversely, when exposed solely to 980 nm light, only PbS QDs absorb photons generating a substantial amount of electron–hole pairs; nevertheless, due to barrier blocking at heterojunctions, only holes can enter into the p-type graphene channel, leading to an increase in carrier concentration and device conductivity.

#### 4.1.3. Schottky Barrier Type

**Figure 8:**
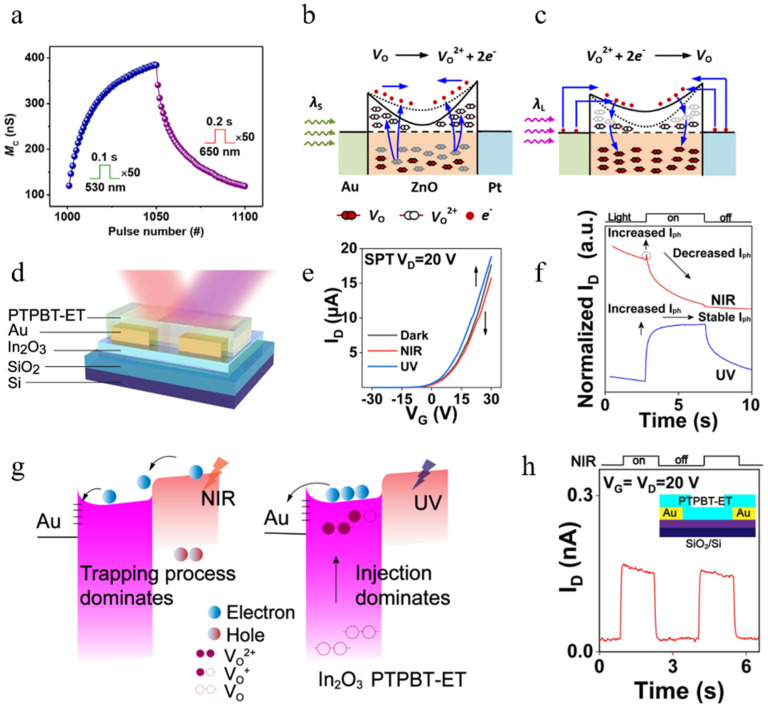
(**a**) Reversible modulation of memconductance can be achieved through the application of 50 pulses of green light, followed by 50 pulses of red light [90]. (**b**,**c**) The energy band diagram of the Pt/ZnO/Au device is depicted after exposure to light with varying wavelengths, including one of shorter wavelength (**b**) and another of longer wavelength (**c**) [90]. (**d**) Illustration of hybrid phototransistor structure [95]. (**e**) Transfer characteristic curves of Schottky contact hybrid phototransistors (SPTs) [95]. (**f**) Normalized currents generated by irradiating SPTs using different types of light [95]. (**g**) Band diagram of the device under NIR and UV irradiation. The gate and drain positions are 20V in both cases [95]. (**h**) The channel current of the bare PTPBT-ET Schottky contact phototransistor under NIR light (2.8 mW/cm^2^, 1.5 s), with VD = VG = 20 V. Inset: Schematic diagram of the device structure [95].

Metal–semiconductor contact can generate a Schottky barrier. Similarly, the band structure at the interface can be adjusted by light to achieve all-optical control. Yang et al. developed an all-optical synaptic device based on an Au/ZnO/Pt structure [90]. Due to ZnO’s lower electron affinity compared to Au and Pt, a Schottky barrier is formed at the interface between the two electrodes and ZnO, as shown in Figure 8b,c for its band structure. The device conductivity changes when subjected to continuous illumination of 50 pulses of 0.1 s duration with 530 nm light (representing SET) and 650 nm light (representing RESET), as shown in Figure 8a. It can be observed that shorter-wavelength light represented by 530 nm induces SET while longer-wavelength light represented by 650 nm induces RESET. The band diagrams depicted in Figure 8b,c explain this phenomenon. When irradiated with short-wavelength light, vacancies within the device become ionized, resulting in numerous positively charged vacancies that cause the downward bending of energy bands, increasing electron tunneling probability and thus enhancing device conductivity. Additionally, under the influence of internal electric fields, electrons generated during vacancy ionization are removed before they can immediately recombine with ionized vacancies; this forms the physical basis for long-term potentiation (LTP) in the device. After exposure to SET light, a large number of ionized vacancies accumulate internally, which can be cleared by RESET light. As shown in Figure 8c, studies have indicated that electrons from metals can be injected into oxides through internal photoelectric or optically assisted tunneling during illumination [93,94]; therefore, when exposed to long-wavelength light, electrons can tunnel from the electrode into the oxide, recombining with ionized oxygen vacancies and increasing the device’s conductivity.

Schottky barriers can also play a unique role in three-terminal devices. Li et al. successfully realized an all-optical synaptic device by selecting the appropriate electrodes and combining the Schottky barrier with a three-terminal device. According to the structure diagram of the device (Figure 8d), it is a Schottky contact hybrid phototransistor (SPTs). Li et al. [95] deliberately chose Au with a large work function as the electrode in order to ensure that the Schottky barrier could be formed with the internal organic/inorganic mixed semiconductor. According to the SPT’s transfer characteristic curve tested in Figure 8e, it can be seen that the larger the gate level is, the larger the current at the drain is. In the case of VG = 20 V, when NIR (near-infrared) light is used to irradiate the device, PSC will be found to decrease, and when UV (ultraviolet) light is used to irradiate, PSC will rise and then stabilize, and the relevant results are shown in Figure 8f. As shown in Figure 8g, Li et al. plotted the energy band diagram of the device. They believe that whether NIR light or UV light irradiation is used, photogenerated electrons will be generated in PTPBT-ET, and because the gate voltage is positive, the electrons will flow into the In_2_O_3_ channel. The difference is that the photogenerated electrons generated by irradiation with UV light have significantly higher energy, and they will cross the Schottky barrier and will not be trapped by the Au and In_2_O_3_ interface. However, the photogenerated electrons generated by using NIR light irradiation cannot cross the Schottky barrier due to insufficient energy, and are eventually trapped by the Au and In_2_O_3_ interface. These trapped electrons will shield the gate voltage and cause PSC drop. To be rigorous, Li et al. fabricated a device without In_2_O_3_ and found that the device was unresponsive to UV light, demonstrating the importance of the Au and In_2_O_3_ interface. It should be noted that the phenomenon of light-assisted tunneling also occurs when short-wavelength light is irradiated. However, due to the presence of a barrier, only a negligible proportion of electrons can tunnel from the metal into ZnO. Additionally, as a large number of free electrons are generated in ZnO and exist in a non-equilibrium state. The quasi-Fermi level shifts towards the bottom of the conduction band and drives electron flow from ZnO into the metal. This further counteracts the electron flow from the metal electrode into ZnO. Therefore, Yang et al.’s all-optical artificial synapse can be considered an artificial synapse based on a “competitive mechanism” [90].

#### 4.1.4. Other Types

Certain devices may possess multiple internal factors that can influence the conductivity of the device. Typically, designers of artificial synapses incorporate two opposing influences into a single device, where the weight of the artificial synapse increases or decreases depending on which influence dominates, known as a “Multi-mechanism” type. There have been numerous papers published on this aspect.

**Figure 9:**
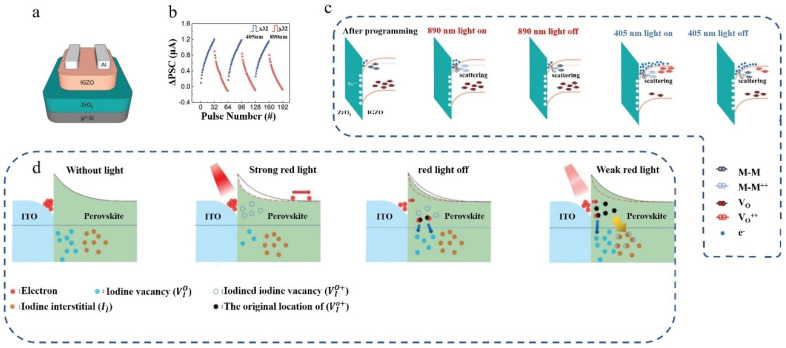
(**a**) The schematic structure of the IGZO/ZrO_x_ photosynaptic transistor [96]. (**b**) Cyclic long-term potentiation and depression properties were observed in the photosynaptic transistor based on IGZO/ZrO_x_ heterojunction [96]. (**c**) Energy band diagrams of the IGZO/ZrO_x_ stack [96]. (**d**) Switching mechanism of the retinomorphic memristor [97].

The conductivity of the device is observed to be dependent on both carrier concentration and mobility. In the proposed all-optical control transistor with IGZO/ZrO_x_ as the functional layer by Mi et al. [96], its structure is illustrated in Figure 9a. Upon exposure to shorter-wavelength light, the PSC initially decreases and then sharply increases. Conversely, when exposed to longer-wavelength light, the PSC decreases until the light is turned off and subsequently increases. Therefore, longer-wavelength light can reduce conductivity while shorter-wavelength light can enhance it. By alternating between long- and short-wavelength irradiation, changes in device conductivity are demonstrated as shown in Figure 9b, along with the corresponding conductance graph. The device contains numerous metal atom pairs with oxygen vacancies (M-M defects), which are distributed at relatively shallow energy levels. Although longer-wavelength light possesses lower photon energy, it is sufficient to excite these defects, resulting in increased carrier scattering and reduced conductivity. Additionally, there are oxygen vacancies (V_oS_) present at deeper energy levels within the device structure. Only short-wavelength light can effectively ionize them. Short-wavelength light can ionize both M-M defects and V_oS_; the former causes the carrier scattering to intensify, and the latter causes the carrier concentration to rise. For this device, the effect of V_oS_ ionization is greater. In the case of long-wave light irradiation, only the M-M defects ionize, which makes the carrier scattering intensify and the device conductance decrease.

Currently, numerous devices employ the mechanism of oxygen vacancy ionization and recombination, wherein the SET light and RESET light exhibit disparate wavelengths. However, in practicality, all-optical synaptic devices can also utilize alternative types of vacancies, with light intensity serving as a controlling parameter. Cai et al. proposed an all-optical memristor based on iodine vacancies [97], as illustrated in Figure 9d. The functional layer of the device comprises Cs_x_FAyMA_1−x−y_Pb(IzBr_1−z_)_3_. When subjected to intense red light irradiation (11.8 mW cm^−2^), its conductivity increases; subsequently subjecting the device to weaker red light (0.9 mW cm^−2^) results in a significantly accelerated decay rate compared to darkness conditions. The band structure of the device is depicted in Figure 9d. Under red light illumination, iodine vacancies ($V_{I}^{o}$) within the device become ionized, while ionized iodine vacancies ($V_{I}^{o+}$) can recombine with interstitial iodine ions ($I_{I}^{-}$) under lighting conditions. With intense illumination, $V_{I}^{o}$ rapidly undergoes ionization, leading to positive charge accumulation at the boundaries, resulting in band bending and increased electron tunneling probability that enhances the conductivity of the device. In dark conditions, $V_{I}^{o+}$ exhibits limited effectiveness for recombination with $I_{I}^{-}$ and only combines with a small number of electrons tunneling from electrodes; hence, conductivity decreases slowly. However, when weak illumination is present, efficient recombination between $V_{I}^{o+}$ and $I_{I}^{-}$ occurs, leading to a significant increase in clearance efficiency for $V_{I}^{o+}$, thereby causing a rapid decrease in conductivity.

### 4.2. Redox Type

The devices discussed earlier in this article predominantly rely on the oxidation and reduction reactions of vacancies (oxygen vacancies, iodine vacancies, etc.), where the oxidation–reduction of vacancies can regulate the curvature of energy bands. However, in reality, oxidation–reduction reactions involve electron gain and loss. As electrons serve as charge carriers, their concentration affects device conductivity. If it is possible to utilize light to control oxidation–reduction processes, then it may be feasible to achieve all-optical control.

#### 4.2.1. Metal Redox Type

**Figure 10:**
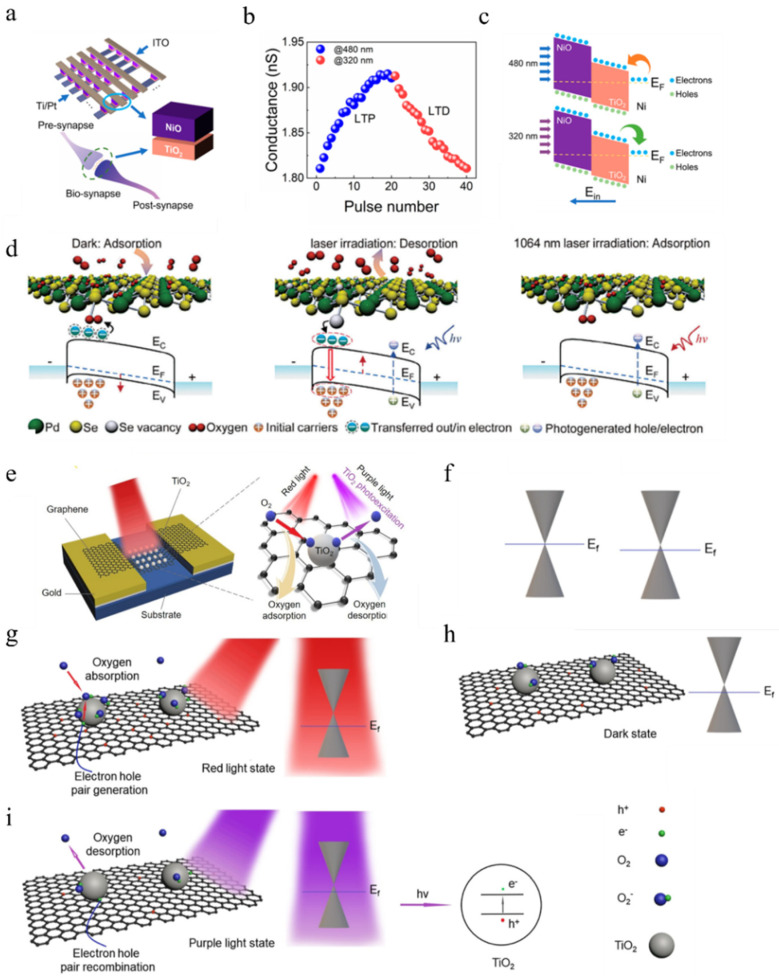
(**a**) The device diagram of the TiO_2_/NiO crossbar array was employed to replicate the functionality of a biosynapse [98]. (**b**) LTP/LTD was fully induced by light at wavelengths of 480 and 320 nm, respectively [98]. (**c**) Device’s energy band diagram when exposed to light stimuli with wavelengths of 480/320 nm [98]. (**d**) Band diagrams of Se-based spacer devices under different illumination conditions [99]. (**e**) Schematic diagram of the Au/TiO_2_ quantum dot–graphene/Au artificial optical synapse [102]. (**f**) Illustration depicting the free energy bands of intrinsic graphene and the energy bands of p-type doped graphene [102]. Illustration showcasing the variation in carrier concentration and energy band of graphene under different conditions, including (**g**) red light state, (**h**) dark state, and (**i**) purple light state [102].

Lu et al. successfully fabricated a resistive switch based on the TiO_2_/NiO heterojunction, which can be fully controlled by light [98]. As illustrated in Figure 10a, the crucial step during device fabrication involved Ni diffusion into TiO_2_ through annealing. Figure 10b demonstrates that the conductivity of the device increases and decreases when illuminated with 480 nm and 320 nm light, respectively. Therefore, in this particular device, longer-wavelength light at 480 nm is associated with SET (switching to a conductive state), while shorter-wavelength light at 320 nm corresponds to RESET (switching back to a non-conductive state). This phenomenon can be explained by the band diagram shown in Figure 10c: when exposed to longer-wavelength light, there is insufficient photon energy to excite electron–hole pairs within NiO and TiO_2_; however, it can oxidize Ni (Ni → Ni^+^ + e^−^), resulting in an increased number of electrons flowing into the conduction band and consequently enhancing device conductivity. Conversely, when exposed to shorter-wavelength light, photons generate electron–hole pairs within both NiO and TiO_2_; some of these electrons will reduce Ni^+^ (Ni^+^ + e^−^ → Ni), leading to a decrease in device conductivity.

#### 4.2.2. Gas Adsorption Analysis Type

The carrier concentration in semiconductors exerts a significant influence on device conductivity. In addition to utilizing light excitation for modulating the carrier concentration, altering the Fermi level position according to the equations n = N_c_exp[−(E_c_ − E_F_)/k_B_T] and p = N_v_exp[−(E_F_ − E_v_)/k_B_T] can effectively modify the carrier concentration. The doping concentration of semiconductors greatly affects the Fermi level, with oxygen being a commonly employed impurity in practical applications. Electrons within devices can transfer to oxygen molecules, resulting in p-type doping and a downward shift of E_F_. Conversely, the desorption of oxygen ions into oxygen molecules leaves behind charges, leading to n-type doping and an upward shift of E_F_. This process can be precisely controlled using light to create all-optical synaptic devices.

Jiang et al. proposed a synaptic device fully controlled by optical means, based on an analysis of oxygen adsorption [99]. By doping AuCl_3_ into PdSe_2_, the channel becomes p-type due to its positive reduction potential. When illuminated with longer-wavelength light (1064 nm), the photocurrent (PSC) increases, while it decreases under shorter-wavelength illumination (473 nm). The band structure of the device is presented in Figure 10d and elucidates the underlying mechanism for this phenomenon. Under dark conditions, Se vacancies attract a significant amount of oxygen, causing channel electrons to transfer to oxygen molecules. With short-wavelength illumination, higher photon energy leads to the desorption of adsorbed oxygen molecules and the exposure of Se vacancies that introduce localized states near the conduction band of PdSe, thereby synergistically inducing n-doping effects in PdSe and increasing E_F_ [100,101], resulting in decreased conductivity. On the other hand, long-wavelength light lacks sufficient energy to desorb oxygen molecules; instead, it merely excites electron–hole pairs, leading to increased conductivity.

Liang et al. proposed a bidirectional all-optical controlled artificial synapse based on graphene/TiO_2_ quantum dots [102], as depicted in Figure 10e. The device’s behavior under different light conditions is illustrated in Figure 10g–i. Red light facilitates the reaction between O_2_ molecules and electrons in the graphene layer through photo-induced molecular adsorption, resulting in the formation of $O_{2}^{-}$ ions and hole accumulation in graphene, leading to a decrease in E_F_. Conversely, purple light generates a significant number of electron–hole pairs in graphene, where holes react with $O_{2}^{-}$ ions to release oxygen and increase E_F_. In the absence of illumination, O_2_ is captured by defects in TiO_2_ and eventually becomes $O_{2}^{-}$, causing p-type doping of graphene. Therefore, a decrease in E_F_ leads to an increase in device conductivity, while an increase in E_F_ without crossing the midline of the bandgap results in decreased conductivity.

## 5. The Material Used to Make Artificial Synapses

### 5.1. The Materials for Manufacturing Optoelectronic Artificial Synapses

There is a wide range of materials available for the fabrication of optoelectronic artificial synapses, each with its unique energy band structure and physical and chemical properties. The selection of appropriate materials for different functional layers plays a crucial role in achieving desired performance. Commonly used materials include various metal oxides, organic compounds, and low-dimensional materials. A variety of materials have been mentioned in review papers [103] as being utilized for the production of artificial optoelectronic synapses.

Each material possesses its unique advantages. Organic materials, for instance, can be processed at low temperatures and in simple solutions, offering a wide variety of options [104]. Moreover, their excellent flexibility enables the fabrication of artificial synapses on these materials (i.e., utilizing organic materials as flexible substrates). Extensive research has been conducted in this area [105,106,107]. Additionally, certain organic materials exhibit high sensitivity to light, rendering them suitable for use as channel materials in optoelectronic synapses [84]. These inherent advantages significantly broaden the scope of applications for organic materials in artificial synapses. Furthermore, it is worth noting that through molecular design, optical and electrical properties can be achieved with organic materials [108], while their molecular structures provide remarkable versatility and flexibility for subsequent processing [109], facilitating a wide range of potential applications. However, compared to inorganic counterparts, the stability of organic materials may be slightly inferior [104], as various physical and chemical processes [110] such as oxygen exposure, moisture ingress, heat exposure, mechanical stress, etc., could potentially lead to the failure of artificial synapses containing organic compounds [111].

Oxides, particularly various metal oxides, find extensive applications in the field of artificial synapse fabrication. In comparison to organic materials, inorganic substances exhibit superior stability. Numerous types of oxides exist, some possessing high mobility [112,113], while others demonstrate light absorption at specific wavelengths [96,114]. Upon appropriate light irradiation, these oxides generate a significant number of photogenerated charge carriers. There are also some oxides that have the advantages of light transmission and good conductivity and are widely used as electrode materials, such as ITO [115]. Furthermore, oxygen vacancies play a pivotal role in numerous artificial synapses where the oxidation and reduction of neutral oxygen vacancies can be employed to regulate energy band curvature [90]. These advantages establish oxygen vacancies as a preferred choice for many artificial synapses.

Low-dimensional materials with at least one dimension at the nanoscale level exhibit distinct properties compared to their bulk counterparts [116]. The integration of these materials with artificial synapses holds great potential for significantly enhancing the quality of such synapses. For instance, two-dimensional halide perovskites possess adjustable molecular structures and bandgaps, enhanced stability in comparison to three-dimensional perovskites, and are not constrained by Goldschmidt’s tolerance factor [117]. This adaptability enables them to be utilized in various applications. The low-dimensional materials can be further categorized into 0-dimensional, 1-dimensional, and 2-dimensional materials, each exhibiting distinct merits and demerits in terms of synthesis complexity, stability, uniformity, etc. This aspect is specifically elucidated in some review articles [116].

### 5.2. The Materials for Manufacturing All-Optical Control Devices

**Figure 11:**
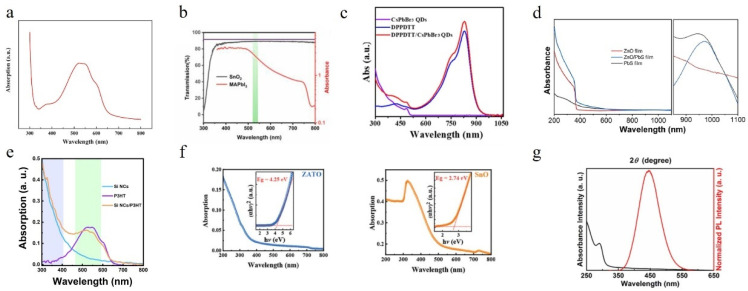
Absorption spectra of some different materials: (**a**) P3HT film [20]; (**b**) MAPbI_3_ film (the transmission spectra of SnO_2_ film can also be seen) [22]; (**c**) CsPbBr_3_ QD film, DPPDTT film, and DPPDTT/CsPbBr_3_ QD hybrid film on quartz [119]; (**d**) ZnO, PbS, and ZnO/PbS films [85]; (**e**) Si NCs, P3HT, and the hybrid Si NC/P3HT film [52]; (**f**) a-ZnAnSnO [43]; (**g**) Cs_3_Cu_2_I_5_ perovskite films [120].

For all-optical synapses, the design of device structures and material selection poses higher requirements due to the need for complex device structures and cumbersome signal modulation processes. Therefore, optimizing materials to enhance device properties has become a prominent research focus [118]. Similar to optoelectronic devices, materials used in optoelectronic devices must exhibit light sensitivity and possess various types. The advantages of different materials align with those of optoelectronic devices and will not be reiterated here. However, unlike optoelectronic devices, some all-optical devices rely on distinct responses from different materials towards varying light stimuli. Figure 11 illustrates the partial absorption spectra of recently reported materials. By carefully selecting appropriate material combinations that enable differential light absorption by distinct functional layers, it becomes feasible to fabricate all-optical control devices.

Other all-optical devices have less stringent requirements for the absorption spectrum and instead prioritize photon energy, focusing on factors such as the bandgap width of semiconductor materials [98], carrier ionization energy at defect levels [96], and the energy needed to desorb oxygen adsorbed on materials [99,102].

## 6. Applications of Optoelectronic All-Optical Synaptic Devices

Due to their unique modulation methods, optoelectronic and all-optical devices have unparalleled advantages in simulating synaptic functions. Thanks to their multidimensional adjustment capabilities, they have promising applications in simulating synaptic plasticity inspired by genetics, optoelectronic and all-optical logic operations, and constructing efficient neural morphological visual systems [121,122]. The following will introduce several important applications.

### 6.1. Brain-like Function Simulation

Numerous studies have demonstrated that the development of artificial synapses to emulate biological synaptic behavior serves as the fundamental basis for constructing efficient neural morphological computing systems [91,123,124]. In optoelectronic devices, it has been widely observed that an increased number of photons and higher energy levels within a given time unit result in a stronger postsynaptic current (PSC) response. This phenomenon has been extensively validated through various articles illustrating STDP, SNDP, SRDP, PPF curves, and index curves [23,81,125,126,127]. However, when considering all-optical synapses, the situation becomes more intricate due to the coordination required between different photosensitive materials with distinct absorption curves. The SET light can be either long-wavelength or short-wavelength light, thus necessitating individual treatment for each specific case. Nevertheless, experimental results should always take precedence. Research indicates that this photo-induced conductance response forms the foundation for simulating higher-order synaptic functions [128].

**Figure 12:**
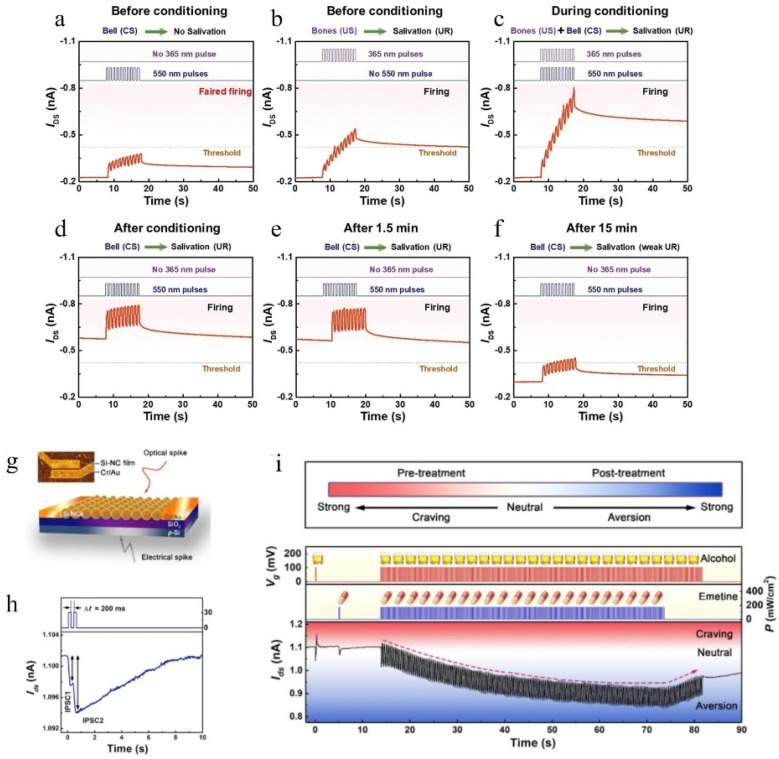
(**a**–**f**) All-optical modulation-based emulation of Pavlov’s associative learning experiment using a broadband optoelectronic synaptic transistor [130]. (**g**) Schematic of a synaptic Si-NC phototransistor [134]. (**h**) PPF and PPD curves of synaptic Si-NC phototransistor [134]. (**i**) Illustration depicting the application of taste aversion learning in addressing alcohol addiction and utilization of synaptic Si-NC phototransistor for taste aversion learning implementation. Schematic of a taste aversion learning process for the treatment of alcoholism and implementation of taste aversion learning with a synaptic Si-NC phototransistor. Electrical stimulation is utilized as a substitute for alcohol, while light stimulation serves as an alternative to emetic drugs [134].

Associative learning is a fundamental cognitive ability of the human brain, and Pavlovian conditioning represents a pivotal experiment in this field [127]. In 2013, O. Bichler et al. developed an organic transistor inspired by Pavlov’s dog experiment [129]. Zhang et al. [130] incorporated PbS quantum dots into PMMA on a Si/SiO_2_ substrate and subsequently formed a heterojunction with Pentacene, successfully fabricating an artificial synapse that mimics the broadband response retina from ultraviolet to near-infrared light. The device effectively demonstrates its capability to simulate Pavlovian associative experiments. Figure 12a–f illustrates the experimental process, wherein EPSC can reach the specified threshold upon exposure to 365nm light but not 550 nm light, indicating that the former corresponds to food stimulus while the latter represents bell stimulus. When both bell and food stimuli are simultaneously applied, it is observed that EPSC still reaches the threshold even after only bell stimulation, akin to how Pavlov’s dog would salivate upon hearing bells after repeated exposure to mixed food and bell stimuli over an extended period of time. However, continuous application of only bell stimulation without food stimulation leads to a decrease in EPSC over time, reflecting the process of forgetting as temporarily established associations decayed [131].

Aversion learning, a well-known form of associative learning in vertebrates, occurs when the taste of food becomes associated with subsequent nausea or other negative consequences [132]. This phenomenon is considered a specific type of learning by researchers [133]. Yin et al. [134] observed that emetic drugs are administered to alcoholics in medical practice to induce vomiting after consuming alcohol, leading to the development of an aversion towards alcohol [135]. Inspired by this concept, they designed a photonic device depicted in Figure 12g. As illustrated in Figure 12h, the application of two consecutive pulses of 1342 nm light resulted in a decrease in PSC, indicating its inhibitory effects. To validate the device’s ability to simulate aversion learning, electrical pulses were defined as alcohol stimuli and optical pulses as emetic drug stimuli; both types were continuously applied to the device. Ultimately, it was found that upon reapplication of electrical pulses, the conductivity remained at a relatively low level simulating an alcoholic’s aversion towards alcohol after treatment. The entire process is illustrated in Figure 12i.

### 6.2. Logical Operations and Arithmetic Operations

The brain, being an intricately complex organ, possesses the remarkable ability to perform intricate calculations [136]. As the fundamental unit of computation, artificial synapses should possess the capability to execute basic logical operations [137] and arithmetic calculations [138], which is pivotal for advancing neuromorphic computing [139]. Certain meticulously designed optoelectronic devices demonstrate the capacity to simulate both logical and algebraic operations.

**Figure 13:**
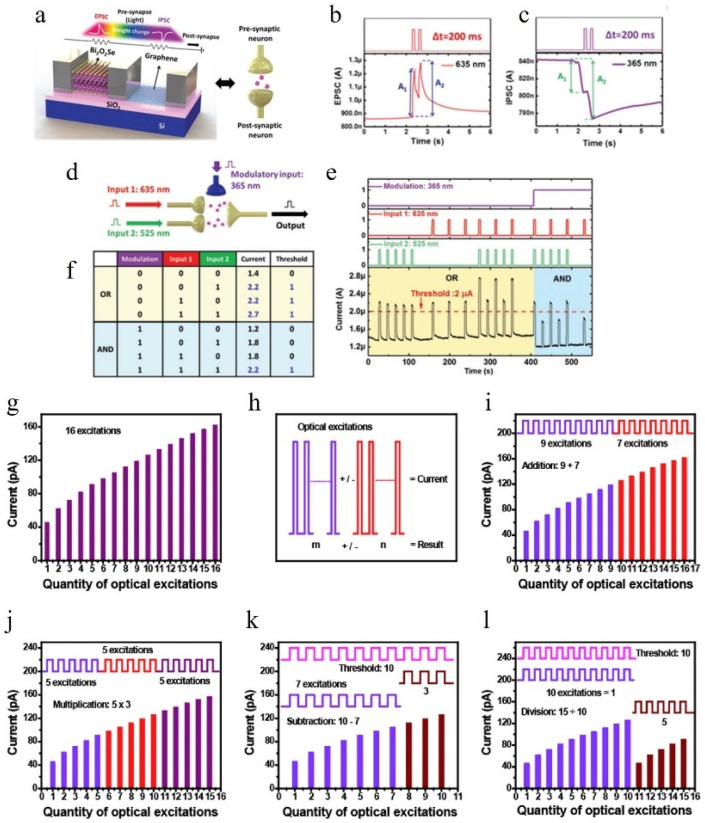
(**a**) Illustration of a biological synapse and an optoelectronic synapse based on Bi_2_O_2_Se/graphene [140]. (**b**,**c**) The alterations in EPSC and IPSC caused by the pair of different optical pulses [140]. (**d**) Schematic operation diagram for the logic gates in a synaptic Bi_2_O_2_Se/graphene photodevice [140]. (**e**,**f**) The output current response over time for various input configurations is depicted. The light and dark representations of each distinct optical input correspond to the binary values 1 and 0, respectively, and (**f**) is the truth table [140]. (**g**) An integer i is assigned in the range of 1 to 16, and the postsynaptic membrane current after the ith light stimulus is denoted as EPSC_i_ [142]. (**h**) Schematic illustration showcasing the functioning of addition and subtraction [142]. (**i**–**l**) Arithmetic operations including addition, multiplication, subtraction, and division [142].

Yang et al. successfully demonstrated logic operations using a hybrid structure of Bi_2_O_2_Se/graphene [140]. The device they fabricated is illustrated in Figure 13a. By irradiating the device with consecutive light sources at 635 nm and 365 nm, the photocurrent response of the device changes as depicted in Figure 13b,c. It can be observed that for this particular device, the short-wavelength light (365 nm) acts as a RESET signal, while the long-wavelength light (635 nm) acts as a SET signal. Based on this principle, Yang et al. were able to simulate OR and AND functions utilizing this device. It should be noted that conventional CMOS circuits achieve logic operations through NMOS and PMOS connections; however, optoelectronic or all-optical synapses heavily rely on their specific structures for functionality. Altering the structure may compromise other synaptic functionalities, making it challenging to modify easily. To address this limitation, Yang et al.’s approach involves introducing a control input terminal, thus increasing fan-in to three for synapses used in two-variable operations (as shown in Figure 13d). The inhibitory effect of 365 nm light on conductivity combined with enhanced conductivity by longer-wavelength light enables the realization of logic operations by selecting an appropriate threshold value for photocurrent, where values exceeding it are defined as ‘1’ and otherwise ‘0’. In fact, for logic operations, inputs do not necessarily have to be limited to light. Tan et al., based on ITO/CeO_2−x_/AlO_y_/Al structures [141], and Zheng et al., based on ZnO/MoO_x_ heterojunction structures [58], have also demonstrated capabilities of logic operation using optoelectronic devices incorporating both light and electricity.

In traditional CMOS circuits, addition necessitates the use of half adders or full adders, while multiplication requires complex multiplication arrays. However, optoelectronic devices offer a promising avenue to simplify computation processes significantly. Specifically, carefully designed devices with excellent linearity demonstrate exceptional performance in algebraic operations. Huang et al. have successfully fabricated an optoelectronic synapse based on an ITO/PCBM/MAPbI_3_:Si NCs/Spiro-OMeTAD/Au structure [142]. Figure 13g–l depict the implementation of addition, subtraction, multiplication, and division using this device. Calculations indicate that this device achieves a Pearson product–moment correlation coefficient of 0.99 and exhibits remarkable linearity. As illustrated in Figure 13i, applying nine light pulses followed by seven light pulses yields an EPSC identical to directly applying sixteen pulses—validating the device’s capability for addition operation. Similarly demonstrated in Figure 13k is the ability to perform subtraction; initially applying seven light pulses and determining the additional number required for EPSC to reach the level achieved with ten light pulse stimulation (which turns out to be three). It has been suggested [143] that multiplication (division) can be implemented similarly to addition (subtraction). Thus, if addition and subtraction are achievable through this approach, then so too are multiplication and division.

### 6.3. Visual Perception System

**Figure 14:**
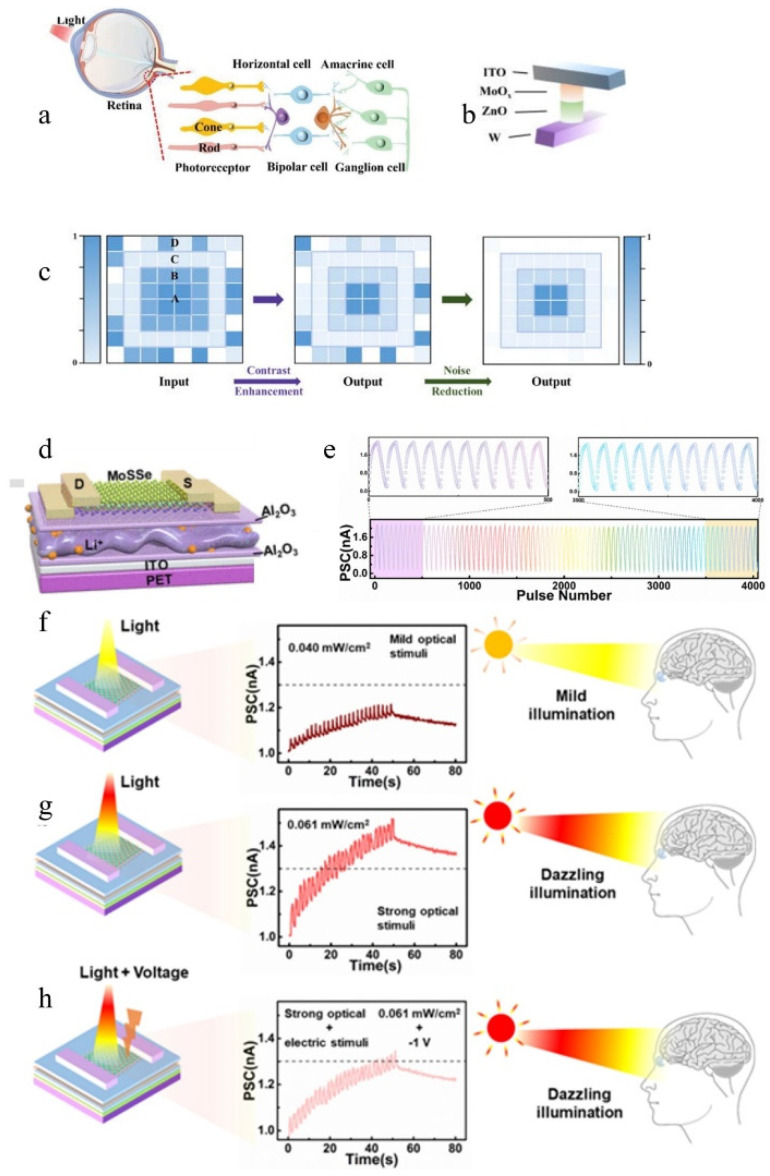
(**a**) Schematic illustration depicting the structure of the human retina [97]. (**b**) Fully light-modulated memristor based on ZnO/MoO_x_ heterojunction for neuromorphic computing [58]. (**c**) Diagram illustrating low-level image preprocessing functions. The middle and right images represent contrast enhancement and noise suppression, respectively, with shadows indicating the magnitude of normalized current, the optical intensities in regions A, B, and C are set at 23.0, 18.4, and 4.6 mW cm^−2^, respectively. Meanwhile, optical signals in region D are designed as random noise, in which the intensities range from 0 to 18.4 mW cm^−2^ [58]. (**d**) Design of 2D Janus MoSSe-based electronic device [152]. (**e**) LTP and LTD were induced by delivering a series of 25 consecutive positive and negative pulses to the gate electrode [152]. (**f**–**h**) In weak light, strong light, and strong light with a −1 V bias, test of the PSC to verify the device’s environmental adaptability [152].

In recent years, many researchers have been inspired by the human visual neural system and have created numerous artificial synapses that can simulate the human visual system. The schematic diagram of the human visual system is shown in Figure 14a.

#### 6.3.1. Image Preprocessing

The human visual system possesses the ability to convert light signals into neural signals. This article presents a comprehensive introduction to numerous all-optical or optoelectronic devices that employ diverse photosensitive materials, thereby exhibiting conductivity modulation in response to incident light. When subjected to an applied voltage, this change in conductivity induces a corresponding alteration in current, akin to the process by which the human visual nervous system transforms light stimulation into neural signals. However, within the visual nervous system, these signals undergo preprocessing and regulation [144], rendering it a focal point for experimental validation across multiple studies. Extensive research has demonstrated that the human visual nervous system effectively transmits information while concurrently filtering noise and enhancing contrast functions [145,146,147]. Zheng et al. developed a fully optically controlled synapse based on the ZnO/MoO_x_ heterojunction [58], as illustrated in Figure 14b. The SET light is ultraviolet (UV), while the RESET light is visible. The right image in Figure 2d demonstrates the variation of photocurrent signal (PSC) under different intensities of UV light illumination for this device, indicating that higher stimulus light intensity leads to slower conductivity decay. To simulate the contrast enhancement and noise reduction functions of human retinas, an 8 × 8 array was constructed, as shown in Figure 14c, where the SET light stimulation intensity decreases from inner to outer rings and random noise is added to the outermost ring. The shadows in Figure 14c represent normalized current, with lighter shades indicating decreased normalized current from inner to outer rings, consistent with input patterns. This demonstrates that this device can effectively mimic the contrast enhancement function of visual neural systems. Furthermore, by utilizing visible light (RESET light), noise can be removed from the outermost ring, simulating the noise reduction function observed in visual neural systems. This research holds significant implications since many applications related to optoelectronic or all-optical synapses are currently focused on constructing artificial neural networks for handwritten digit or face recognition [126,148]. Lower image noise results in improved training outcomes [149,150]. Compared with traditional denoising algorithms, synaptic denoising may find potential applications in fast low-power scenarios.

#### 6.3.2. Environmental Adaptability

In the previous discussions, there was no mention of the current tolerance of the device. In reality, any device has a maximum operating current limit. Sudden intense exposure to bright light can cause an impulse current that may damage the functional layers of the device and accelerate its aging. In the real biological visual neural system, there are a series of complex mechanisms to prevent organisms from being damaged by excessive stimulation [151].

Meng et al. developed an environmentally adaptive device based on 2D Janus MoSS [152], as depicted in Figure 14d. The device underwent 25 consecutive positive and negative pulses applied to the gate, with multiple cycles performed, as shown in Figure 14e. It can be observed that the device demonstrates excellent durability, and applying negative pulses to the gate effectively suppresses EPSC. A threshold current is defined to indicate intense light stimulation and eye discomfort when the PSC exceeds this threshold, as illustrated in Figure 14g. Under identical light stimulation conditions as depicted in Figure 14h, applying a −1 V voltage to the gate reduces the PSC below the threshold level, indicating synaptic adaptation to environmental changes. In addition to individual devices, Kwon et al. also constructed a simple circuit using multiple transistors and achieved exceptional perception behavior of environmental adaptability under various illumination levels by adjusting the load transistor within the circuit [36].

### 6.4. Artificial Neural Network

**Figure 15:**
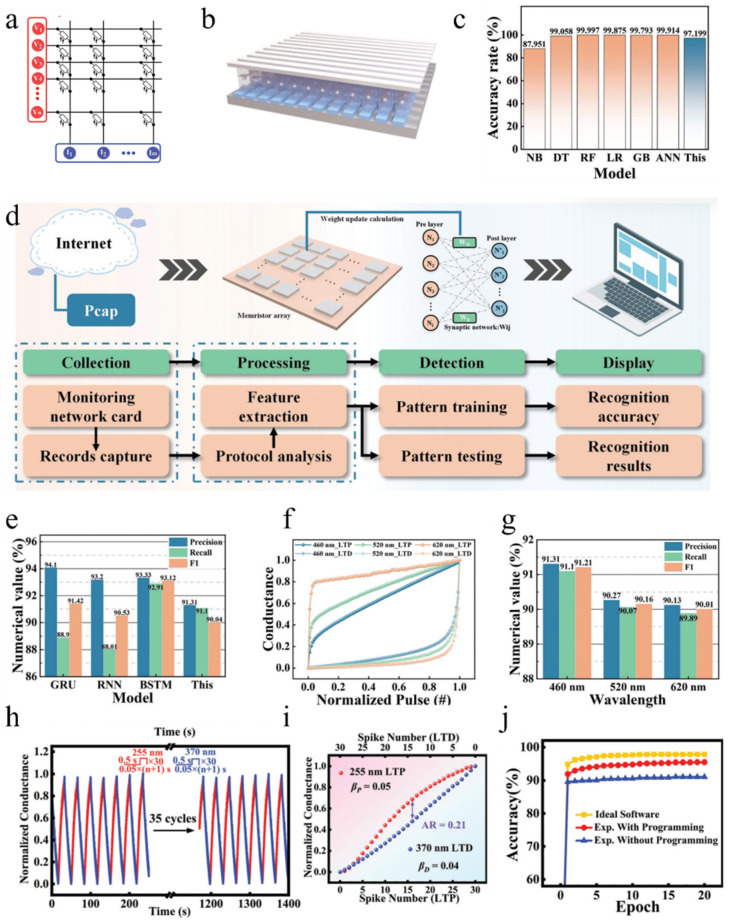
(**a**) Artificial neural networks using artificial synapses [155]. (**b**) Schematic diagram of neural network based on WO_3−X_/WO_3−X_-Ag/WO_3−X_ optoelectronic memristor [156]. (**c**) Evaluation of the identification outcomes obtained from various neural network algorithms [156]. (**d**) Flowchart of IDS [156]. (**e**) Comparison of the results for the different algorithms [156]. (**f**) The conductance values normalized for different light modulation wavelengths [156]. (**g**) The evaluation metrics, including precision, recall, and F1, were computed for the models that utilized varying light wavelengths to modulate the conductivity values’ weights [156]. (**h**) PSC generated through the application of UV pulses at wavelengths of 255 nm and 370 nm, as well as (**i**) the correlation between normalized conductance and the quantity of LTP/LTD spikes during programming duration [155]. (**j**) Handwritten digit recognition results under different circumstances [155].

Currently, the fourth industrial revolution—the revolution of intelligence—has swept across the globe. In the era of artificial intelligence, excellent algorithms and powerful computing power have become the foundation for development. In recent years, with the rise of deep learning, the application of large-scale neural networks has greatly enhanced the performance of artificial intelligence in recognition tasks. However, its massive amount of data poses high demands on hardware devices [153]. In terms of architecture, the von Neumann architecture consumes a significant amount of time and energy due to its separation of storage and computation, resulting in data transportation. From the perspective of individual devices, Moore’s Law has reached its limit with shrinking feature sizes, making it increasingly expensive to further reduce device dimensions and hindering product popularization. The use of an integrated storage–computation architecture holds promise in addressing these issues; however, using CMOS devices for neuromorphic computing chips requires complex peripheral circuits that may offset the benefits brought by the integrated architecture. Therefore, we need new devices to replace CMOS devices [154] in order to fully exploit the potential of integrated storage–computation architectures, with artificial synapses being a highly promising candidate. Figure 15a illustrates an artificial neural network (ANN) constructed using artificial synapses [155], where the synaptic weights can be reflected by the conductance of the devices between horizontal and vertical bars. The following section will discuss the advantages of using optoelectronic devices for constructing neural networks from a power consumption perspective and highlight ways to improve recognition accuracy in deep networks.

#### 6.4.1. Low-Power Neural Networks

Yang et al. [156] developed a photoelectric memristor based on WO_3−x_/WO_3−x_-Ag/WO_3−x_, which successfully achieved STP, LTP, and other functions. They used this device to construct a convolutional neural network based on a memristor array (M-CNN), as shown in Figure 15b. The KDDCup-99 dataset contains some numbers and strings, which Yang et al. converted into an 8 × 8 numerical feature matrix. After conducting 494,021 training iterations, they evaluated the accuracy of the training using 311,029 test data points and found that it reached 97.2%. Compared to other neural network algorithms based on traditional hardware recognition methods, the recognition progress for the KDDCup-99 dataset was slightly higher but consumed much more energy than M-CNN (which only consumed 10^−6^ W power during training). Subsequently, Yang et al. built an intrusion detection system (IDS) that integrated real-time network data collection, processing, and detection capabilities to verify its ability to detect abnormal network records in the KDDCup-99 dataset. The flowchart of IDS is shown in Figure 15d. Precision, recall, and F1 were used as parameters to evaluate the performance of the system; higher values indicate better performance for all three parameters. Random samples from various classes totaling 41,847 were selected from the KDDCup-99 dataset for testing purposes and compared with results obtained using other traditional algorithms, as shown in Figure 15e. Although M-CNN-based IDS had a smaller F1 parameter value compared to others considered here, its extremely low power consumption level makes it acceptable.

#### 6.4.2. Improved Accuracy through Varying the Light Stimulus

Many studies have shown that the linearity and symmetry of device conductivity increase and decrease are crucial for the accuracy of artificial neural network recognition [38]. Here, we mainly introduce how to make the device conductivity increase and decrease symmetrically and linearly by applying appropriate light stimulation. Yang et al. [156] fully utilized the advantage of their devices having a wide bandwidth and tried various wavelengths of light. Figure 15f shows the conductivity increase and decrease curves under three different experimental light modulations, with 460 nm light showing the best linearity and symmetry. It can be anticipated that using 460 nm light stimulation will maximize precision, recall, and F1 parameters, which is consistent with the results shown in Figure 15g. Sun et al. [155] fabricated an all-optical control device based on Si-doped beta-gallium oxide (*β*-Ga_2_O_3_)/ZnO heterojunctions. They found that when irradiating the device with SET (RESET) light, the normalized conductivity increase (decrease) trend is initially fast then slows down, resembling a logarithmic curve overall. In order to obtain better symmetry and linearity in the curve, they defined that the width D of optical stimulation has a functional relationship with nth optical stimulation as: D = 0.05 × (n + 1). As seen from Figure 15h,i, both the linearity and symmetry of conductance change have been greatly improved. Figure 15j shows the accuracy of handwritten font recognition under ideal conditions (yellow curve), uniform optical pulse stimulation (blue curve), and non-uniform optical pulse stimulation (red curve). It can be observed that customized planning of optical stimuli for different devices is very helpful in improving efficiency in artificial neural network recognition.

## 7. Summary and Outlook

The limitations of traditional von Neumann computer architecture are increasingly amplified, while optoelectronic devices and all-optical devices have been extensively investigated in recent years as promising candidates with aspirations for chip-level brain-like computation. In this article, we present an introduction to the fundamental functions of neural synapse devices such as long-term potentiation, short-term potentiation, paired-pulse facilitation, learning mechanisms, and forgetting processes within learning models. At the same time, the basic principles of optoelectronic and all-optical synapses are introduced in the third and fourth parts, respectively. In the fifth part, various materials for fabricating optoelectronic and all-optical artificial synapses are introduced. In the sixth part, their applications in brain-like functional simulation, logical and algebraic operations, visual nervous systems, and low-power neural networks are introduced. In the sixth part, their applications in brain-like functional simulation, logical and algebraic operations, visual nervous system and low-power neural network are introduced.

The field of optoelectronic artificial synapses and all-optical artificial synapses holds promising prospects, yet due to their relatively short research history of less than a decade, the exploration is still in its nascent stages. Therefore, we present the following future perspectives:

(I) Synapses represent only a fraction of nerve cells. Presently, research is solely focused on simulating synapses rather than the entire neuron. Other components of nerve cells, such as axons and dendrites, also possess significant research value. Dendrites receive information from other cells, then transmit the information to the axon, which in turn connects with dendrites of other neurons to pass on the information. In previous discussions, the focus has been on studying the properties of individual synapses. In subsequent research, integrating multiple neurons to perform more complex tasks, especially simulating the process of information transmission in the human brain, will become a research hotspot.
(II) In the post-Moore’s Law era, there has been a remarkable surge in transistor density on chips and processor operating frequency, resulting in a substantial increase in power consumption. Although artificial synapses hold promise for addressing this challenge, their complete potential remains largely unexplored. RRAM (resistance random access memory) devices can mimic the function of biological synapses through their electrical properties. The relevant literature indicates that RRAM’s theoretical minimum cell area can reach at least 4F^2^, where F represents the feature size of a given process [157]; however, few authors have addressed the issue of device size optimization in their papers. On the other hand, regarding the problem of applying the stimulus, take the device shown in Figure 10a as an example. In this paper, the authors apply SET light and RESET light for up to 1000 s to make the device conductance rise and fall. According to “Work is equal to power times time”, the electrical work consumed will increase with time. Even if the PSC power is not high, it will cause unnecessary energy consumption. In addition, the energy of the light source will also be consumed in this process. Moreover, the exposure time of 1000 s also proves that the device is not suitable for the situation where fast reaction is required. One possible approach could involve reducing device size and excitation light width; however, these strategies impose greater demands on manufacturing processes and synaptic performance [33].
(III) In practical situations, the tasks that can be accomplished by a single synapse are very limited, so integration will become a hot topic in future research. The cooperation of multiple devices will result in better system performance. However, dual-terminal devices often face the obstacle of crosstalk between adjacent devices due to their higher integration density. On the other hand, three-terminal devices can handle more complex tasks due to gate control. Therefore, trade-offs need to be made based on applications in practical situations. Taking the crossbar structure shown in Figure 15a as an example, devices are placed at the intersection of both the transverse and longitudinal crossbars. If you only want to use the crossbar structure to complete some computational tasks (such as matrix multiplication operation), then you can use two-terminal devices to form the crossbar structure. At present, there have been reports in this aspect [158]. In addition, there are also reports that the combination of two-terminal and three-terminal devices is used to form a one-transistor-one-memristor (1T1R) structure. Yao et al. built a complete five-layer convolutional neural networks for digital image recognition based on the crossbar structure of 1T1R, with a training accuracy of 96.19% and a 3-fold reduction in latency [159].
(IV) Durability and manufacturability are crucial for a device to be manufactured as a product. Durability and manufacturability encompass three aspects. Firstly, the device’s performance must remain stable after undergoing numerous SET and RESET processes. Secondly, it should retain its properties over an extended period of time, regardless of different environmental conditions. Thirdly, the manufacturing process must be capable of consistently producing devices with uniform properties. Achieving durability poses a significant challenge. If we define the change in conductance (or PSC) from the initial value to the maximum value and back to the initial value as one cycle, then it can be observed that the device depicted in Figure 9a has undergone three cycles. As illustrated in Figure 9b, the PSC–pulse number curves obtained during these three cycles exhibit a high degree of similarity, indicating an ideal scenario. Similar experiments have been reported in multiple articles [58,90]. A perfect device is one for which, no matter how many cycles it has gone through, the PSC–pulse number curve is basically overlapping, which has been reported in many articles at present. However, the tremendous success of CMOS devices has inspired us to conduct the following experiments: ① Whether the properties of the devices degrade significantly after long-term placement (especially in extreme environments such as humidity and high temperature). ② How much deviation is there in devices produced with the same process? This experiment is very important because it determines whether a standard manufacturing process can be developed.
(V) Artificial synapses are an interdisciplinary field that requires knowledge from physics, chemistry, and biology. Basic science serves as the foundation for all sciences; therefore, researchers working on optoelectronics or all-optical studies should always keep track of advancements in these three domains. Artificial synapses essentially mimic biological systems where biology provides theoretical foundations, while chemistry and physics offer materials for implementation and new principles.

For various reasons, we will find that in the real world, even though artificial synapses have better performance than traditional CMOS devices and have great potential to break the von Neumann memory wall, we have to admit that CMOS devices still occupy the market. Artificial synapse bionic accuracy is not high enough. The circuit function implementation is relatively elementary (mostly used as hardware accelerator for artificial deep neural network algorithm), and the integration level is not high enough. In order to achieve higher requirements, it is often necessary to cooperate with traditional transistors. The fabrication of artificial synapses using conventional processes used to fabricate CMOS integrated circuits (such as chemical vapor deposition, sputtering, etc.) introduces a range of randomness in device properties. However, it is reasonable to believe that with the development of new materials and new device structures, the application of artificial synapses will eventually promote the performance of AI to an unprecedented level.
